# SIRT6 Is Involved in the Progression of Ovarian Carcinomas via β-Catenin-Mediated Epithelial to Mesenchymal Transition

**DOI:** 10.3389/fonc.2018.00538

**Published:** 2018-11-20

**Authors:** Jun Sang Bae, Sang Jae Noh, Kyoung Min Kim, See-Hyoung Park, Usama Khamis Hussein, Ho Sung Park, Byung-Hyun Park, Sang Hoon Ha, Ho Lee, Myoung Ja Chung, Woo Sung Moon, Dong Hyu Cho, Kyu Yun Jang

**Affiliations:** ^1^Department of Pathology, Chonbuk National University Medical School, Chonbuk National University, Jeonju, South Korea; ^2^Biomedical Research Institute, Chonbuk National University Hospital, Jeonju, South Korea; ^3^Research Institute for Clinical Medicine, Chonbuk National University, Jeonju, South Korea; ^4^Department of Forensic Medicine, Chonbuk National University Medical School, Chonbuk National University, Jeonju, South Korea; ^5^Department of Bio and Chemical Engineering, Hongik University, Sejong, South Korea; ^6^Faculty of Science, Beni-Suef University, Beni Suef, Egypt; ^7^Department of Biochemistry, Chonbuk National University Medical School, Chonbuk National University, Jeonju, South Korea; ^8^Division of Biotechnology, Chonbuk National University, Iksan, South Korea; ^9^Department of Obstetrics and Gynecology, Chonbuk National University Medical School, Chonbuk National University, Jeonju, South Korea; ^10^Research Institute for Endocrine Sciences, Chonbuk National University, Jeonju, South Korea

**Keywords:** ovary, carcinoma, SIRT6, β-catenin, prognosis

## Abstract

SIRT6 is involved in various cellular signaling pathways including those involved in tumorigenesis in association with β-catenin. However, the role of SIRT6 in tumorigenesis has been controversially reported and the studies on the role of SIRT6 in ovarian cancers is limited. In this study, we evaluated the expression and roles of SIRT6 in conjunction with the expression of active β-catenin in 104 human ovarian carcinomas and ovarian cancer cells. In human ovarian carcinomas, the expressions of SIRT6 and active β-catenin were associated with higher tumor stage, higher histologic grade, and platinum-resistance. Moreover, nuclear expression of SIRT6 (104 ovarian carcinomas; *P* = 0.010, 63 high-grade serous carcinomas; *P* = 0.040), and activated β-catenin (104 ovarian carcinomas; *P* = 0.013, 63 high-grade serous carcinomas; *P* = 0.005) were independent indicators of shorter overall survival of ovarian carcinoma patients in multivariate analysis. In OVCAR3 and OVCAR5 ovarian cancer cells, knock-down of SIRT6 significantly inhibited the migration and invasion of cells, but did not inhibit the proliferation of cells. SIRT6-mediated invasiveness of ovarian cancer cells was associated with the expression of epithelial-to-mesenchymal transition-related signaling molecules such as snail, vimentin, N-cadherin, E-cadherin, and activated β-catenin. Especially, SIRT6-mediated increase of invasiveness and activation of epithelial-to-mesenchymal transition signaling was attenuated by knock-down of β-catenin. In conclusion, this study suggests that SIRT6-β-catenin signaling is involved in the epithelial-to-mesenchymal transition of ovarian cancer cells, and the expression of SIRT6 and active β-catenin might be used as indicators of poor prognosis of ovarian carcinoma patients. In addition, our results suggest that SIRT6-β-catenin signaling might be a new therapeutic target of ovarian carcinomas.

## Introduction

SIRT6 is a member of the sirtuin family and is involved in aging, metabolism, DNA damage repair, cell cycle regulation, apoptosis, and epithelial-to-mesenchymal transition (EMT) (1–6). Especially, SIRT6 is protective for various human diseases such as diabetes mellitus (7, 8), degenerative neural disease (9), hepatic ischemic reperfusion injury (10), idiopathic pulmonary fibrosis (3), and muscular atrophy (11). In addition, SIRT6 has been suggested as a tumor suppressor because loss of SIRT6 is associated with increased tumor formation and shorter survival of cancer patients (12, 13). Increased expression of SIRT6 is associated with favorable prognosis of gastric and pancreatic cancer patients (14, 15). However, an oncogenic role of SIRT6 also has been reported in various human cancers. SIRT6 is involved in tumor progression by promoting cell cycle progression and tumor growth (16, 17), inhibiting apoptosis (18), and enhancing EMT-related invasiveness of cancer cells (19, 20). In addition, SIRT6 expression is higher in cancer tissue compared with normal tissue in esophagus, thyroid, and melanocytes (17, 18, 21). Moreover, SIRT6 expression is associated with poor prognosis of breast, gastric, colorectal, and lung cancer patients (19, 22–24), and higher SIRT6 expression is associated with chemoresistance (25, 26). Therefore, a careful approach and further study is needed to explore the role of SIRT6 in human cancers.

Ovarian cancer represents ~4% of cancers in women. However, the fatality rate of ovarian cancer is relatively higher than other cancers of female reproductive organs and is the eighth cause of cancer death in women (27). Although, there has been a continual improvement in survival of ovarian cancer patients in association with recent advances in targeted therapies (27), the survival of advanced ovarian cancer patients is limited, and the 5-year survival rate of ovarian cancer patients with distant metastasis is only 28.9%. Therefore, further study is needed to improve the therapeutic efficacy of advanced ovarian cancer patients. When considering the diverse roles of SIRT6 in cell biology and tumorigenesis, SIRT6 might be a potential therapeutic target in ovarian cancers (1, 6, 12, 13, 17, 18, 22, 24). However, despite extensive studies regarding the role of SIRT6 in human cancers, there has been no report investigating the role of SIRT6 in ovarian cancers. In addition, in breast cancers, one of the most common cancers in women, the role of SIRT6 has been controversially reported (28, 29). As one possible mechanism of the oncogenic role of SIRT6, we previously presented the role of the CK2α-SIRT6-β-catenin pathway in the progression of breast carcinomas (22). Therefore, based on our previous study, we investigated the expression patterns and roles of SIRT6 and active (de-phosphorylated) β-catenin in human ovarian carcinomas and ovarian cancer cells.

## Materials and methods

### Ovarian carcinoma patients and tissue samples

We evaluated one hundred and four ovarian carcinomas which were used in our previous study (30). The patients underwent therapeutic operations between November 1996 and August 2008. The clinicopathological information was obtained from patients' medical records. The ovarian carcinomas were classified according to the World Health Organization classification (31) and staged according to the American Joint Committee on Cancer staging system (32). Cancer tissues included 75 serous carcinomas, 20 mucinous carcinomas, 5 endometrioid carcinomas, 3 clear cell carcinomas, and 1 malignant Brenner tumor. The ovarian carcinomas were grouped according to following clinicopathological factors: age (< 60 years vs. ≥60 years), pre-operative serum level of CA125 (reference value; 0–35 U/ml, normal vs. elevated,), tumor stage (I & II vs. III & IV), tumor size (≤10 cm vs. >10 cm), lymph node metastasis (absence vs. presence), ascites (absence vs. presence), bilaterality (unilateral vs. bilateral), histologic grade (low; grade 1 vs. high; grade 2 and 3), and platinum resistance (absence *vs*. presence). Among the 83 patients who received platinum- and taxoid-based adjuvant chemotherapy, 62 patients were platinum-sensitive, 20 patients were platinum-resistant, and 1 patient was not evaluable for platinum resistance according to the standard Gynecologic Oncology Group criteria (33). The duration of follow-up ranged from 1 to 209 months (median; 82 months). This study obtained approval from the institutional review board of Chonbuk National University Hospital (IRB number, CUH 2017-12-016) and was performed according to the Declaration of Helsinki. The approval contained a waiver for written informed consent based on the retrospective and anonymous character of the study.

### Immunohistochemical staining in tissue sections

Immunohistochemical staining was performed on tissue microarray sections. The diameter of each tissue microarray core was 5 mm and one core per case was constructed in the area of the highest histologic grade. Antigen retrieval was performed by boiling in a microwave oven in pH 6.0 antigen retrieval solution (DAKO, Glostrup, Denmark) for 20 min. Anti-SIRT6 (Cell Signaling Technology, Beverly, MA) and anti-active β-catenin (Millipore, Darmstadt, Germany) antibodies were used as primary antibodies. The stained slides were scored by two pathologists (KJ and SN) with consensus without knowledge of the clinicopathological information. The scoring for the immunohistochemical expression of SIRT6 and active β-catenin were performed according to the Allred scoring system (8, 34, 35). The clinical significance of SIRT6 and active β-catenin expression patterns might differ according to their cytoplasmic or nuclear expressions (22, 28); therefore, we separately analyzed SIRT6 and active β-catenin according to their cytoplasmic and nuclear expression patterns. The immunohistochemical scores were obtained by adding the staining intensity score (0; no staining, 1; weak, 2; intermediate, 3; strong) and the staining area (0; no staining, 1; 1%, 2; 2–10%, 3; 11–33%, 4; 34–66%, 5; 67–100%) (8, 34, 35). Therefore, the immunohistochemical scores ranged from zero to eight.

### Ovarian cancer cells and cell culture

In this study, we used two human ovarian cancer cell lines. OVCAR3 cells were purchased from the Korean Cell Line Bank (KCLB, Seoul, Korea) and OVCAR5 cells were purchased from the Cell Biolabs Inc. (Cell Biolabs Inc., San Diego, CA). The OVCAR3 and OVCAR5 cells were cultured in RPMI 1640 and DMEM medium, respectively. Culture media contained 10% fetal bovine serum and penicillin/streptomycin (100 U/ml) (Gibco BRL, Gaithersburg, MD). The cells were cultured in a humidified 5% CO_2_ incubator at 37°C.

### Plasmids and transfection

SIRT6-specific shRNA expression vector was purchased from GenePharma Co. (GenePharma, Shanghai, China). The SIRT6 duplex had the sense and antisense sequences 5′-CACCGCTACGTTGACGAGGTCATGATTCAAGAGATCATGACCTCGTCAACGTAGCTTTTTTG-3′ and 5′-GATCCAAAAAAGCTACGTTGACGAGGTCATGATCTCTTGAATCATGACCTCGTCAACGTAGC-3′, respectively. A pFLAG-CMV-2 plasmid vector was used as a control vector. The plasmid for wild-type SIRT6 (pFLAG2_SIRT6) was synthesized by Cosmogenetech Co. Ltd. (Seoul, Korea). The shRNA expression vector for β-catenin was purchased from Santa Cruz Biotechnology (# sc-29209-SH, Santa Cruz, CA). Lipofectamine 2000 (Invitrogen, Carlsbad, CA) was used for transfection.

### Cell proliferation assay and colony forming assay

The proliferation of cells by counting the number of cell, a 3-(4,5-dimethylthiazol−2-yl)-2,5-diphenyltetrazonium bromide (MTT) (Sigma-Aldrich, St. Louis, MO) assay, bromodeoxyuridine (BrdU) incorporation assay (Roche Applied Science, Mannheim, Germany), and a colony-forming assay. For determining the cell number in the proliferation assay, OVCAR3 (2 × 10^3^) and OVCAR5 (2 × 10^3^) cells were seeded in 24-well plates and the number of viable cells was counted at the indicated time points. For the MTT assay, OVCAR3 (2 × 10^3^) and OVCAR5 (1 × 10^3^) cells were seeded in 96-well culture plates. Absorbance was measured at 560 nm using a microtiter plate reader (Bio-Rad, Richmond, CA). The BrdU incorporation assay was performed by seeding OVCAR3 (2 × 10^3^) and OVCAR5 (1 × 10^3^) cells in 96-well plates. Incorporated BrdU was measured with a microtiter plate reader (Bio-Rad, Richmond, CA) at a wavelength of 370 nm. For the colony-forming assay, OVCAR3 (4 × 10^3^) and OVCAR5 (2 × 10^3^) cells were cultured in 6-well culture plates for 10 days. At day 10, the colonies were fixed with methanol and stained with methylene blue.

### Wound healing assay and *in vitro* migration and invasion assays

For the wound healing assay, OVCAR3 and OVCAR5 cells were seeded in 60 mm culture dishes and grown to 100% confluency. Thereafter, a linear wound was made on the cells using a 200 μl pipette tip and microscopic images were taken at that time and 24 h after wound generation. The migration assay was performed with a 24-transwell chamber (Corning Life Sciences, Acton, MA). For the migration assay, OVCAR3 (1 × 10^5^) and OVCAR5 (5 × 10^4^) cells in serum-free culture medium were seeded into the upper chambers, and media with 20% fetal bovine serum was added to the lower chamber as a chemoattractant. An invasion assay was performed with a bioCoat Matrigel Invasion chamber (BD Biosciences, San Jose, CA). For the invasion assay, OVCAR3 (2 × 10^5^) and OVCAR5 (1 × 10^5^) cells in culture media with 2% fetal bovine serum were seeded in a Matrigel-coated upper chamber. The lower chamber was filled with media containing 20% serum as a chemoattractant. The migration and invasion chambers were incubated for 24 h and stained with a Diff-Quick solution kit. The number of cells which had migrated or invaded the chambers were counted in five microscopic fields (magnification × 100) per well.

### Western blot analysis

Total protein was obtained by lysing the cells with PRO-PREP Protein Extraction Solution (iNtRON Biotechnology Inc., Korea) and 1 × phosphatase inhibitor cocktails 2, 3 (Sigma-Aldrich). The primary antibodies used in western blot were as follows: SIRT6 (Cell Signaling Technology, Beverly, MA), snail (Abcam, Cambridge, UK), vimentin (Santa Cruz Biotechnology, Santa Cruz, CA), MMP9 (Thermo Fisher Scientific, Fremont, CA), MMP2 (R&D Systems, Minneapolis, MN), β-catenin (BD Biosciences), active β-catenin (Millipore, Darmstadt, Germany), E-cadherin (BD Biosciences), N-cadherin (BD Biosciences), and actin (Sigma-Aldrich). The proteins were detected by a LAS-3000 luminescent image analyzer (Fuji Film, Tokyo, Japan). The results of the western blot were digitally quantified using ImageJ software (ImageJ, version 1.38e, NIH, Bethesda, MD).

### Quantitative reverse-transcription polymerase chain reaction

RNA was obtained with an RNeasy Mini Kit (Qiagen Sciences, Valencia, CA). Reverse transcription of 1.5 μg RNA was performed with Taqman Reverse Transcription Reagents (Applied Biosystems, Foster City, CA). Applied Biosystems Prism 7900HT Sequence Detection System and SYBR Green polymerase chain reaction Master Mix (Applied Biosystems) were used for quantitative reverse-transcription polymerase chain reaction. All experiments were performed in triplicate, and the values were normalized to the expression of the glyceraldehyde-3-phosphate dehydrogenase reference housekeeping gene. The sequences of the primers used in quantitative reverse-transcription polymerase chain reaction are listed in Table 1.

**Table 1:** Primer sequences used for quantitative real-time polymerase chain reaction.

**Gene**	**Primer sequence Forward/Reverse**	**Product size**	**Accession number**
*SIRT6*	F: 5′-AGGATGTCGGTGAATTACGC-3′ R: 5′-AAAGGTGGTGTCGAACTTGG-3′	261	NM_016539.2
*CTNNB1*(β-catenin)	F: 5′-AAAATGGCAGTGCGTTTAG-3′ R: 5′-TTTGAAGGCAGTCTGTCGTA-3′	100	NM_001904.3
*SNAIL*	F: 5′-GCACATCCGAAGCCACAC-3′ R: 5′-GGAGAAGGTCCGAGCACAC-3′	225	NM_005985.3
*Vimentin*	F: 5′-TACAGGAAGCTGCTGGAAGG-3′ R: 5′-ACCAGAGGGAGTGAATCCAG-3′	104	NM_003380.4
*N-cadherin*	F: 5′-ACAGTGGCCACCTACAAAGG-3′ R: 5′-CCGAGATGGGGTTGATAATG-3′	201	NM_001792.4
*E-cadherin*	F: 5′-CCCGGGACAACGTTTATTAC-3′ R: 5′-GCTGGCTCAAGTCAAAGTCC-3′	72	NM_004360.3
*MMP9*	F: 5′-GACGCAGACATCGTCATCCA-3′ R: 5′-GCCGCGCCATCTGCGTTTCCAAA-3′	200	NM_004994.2
*MMP2*	F: 5′-CGGCCGCAGTGACGGAA-3′ R: 5′-CATCCTGGGACAGACGGAAGTTCTT-3′	212	NM_004530.4
*GAPDH*	F: 5′-AACAGCGACACCCACTCCTC-3′ R: 5′-GGAGGGGAGATTCAGTGTGGT-3′	258	NM_001256799.1

*Web link to accession numbers: https://www.ncbi.nlm.nih.gov/gene*.

### Statistical analysis

The cut-off points for the immunohistochemical scores of SIRT6 and β-catenin were determined by receiver operating characteristic curve analysis. The survival analysis was performed for overall survival (OS) and relapse-free survival (RFS). The endpoint of follow-up was the date of last contact or date of death of patients through December 2014. The duration of OS analysis was calculated as the time from the date of diagnosis to the date of last contact or death of patients. The events in OS analysis were death of the patients from ovarian carcinomas. The duration of RFS was calculated from the date of diagnosis to the date of last contact, relapse, or death of patients. The events in RFS analysis were relapse or death of patients from ovarian carcinomas. Survival analysis was performed with univariate and multivariate Cox proportional hazard regression analysis, and Kaplan-Meier survival analysis. The associations between the clinicopathological factors were analyzed using Pearson's chi-square test and the student's *t*-test. SPSS software (version 20.0) was used in statistical analysis. The data was expressed as mean ± standard deviation and *P-*values < 0.05 were considered statistically significant.

## Results

### The expression of SIRT6 and active β-catenin are associated with advanced clinicopathological factors of ovarian carcinoma patients

**Figure 1:**
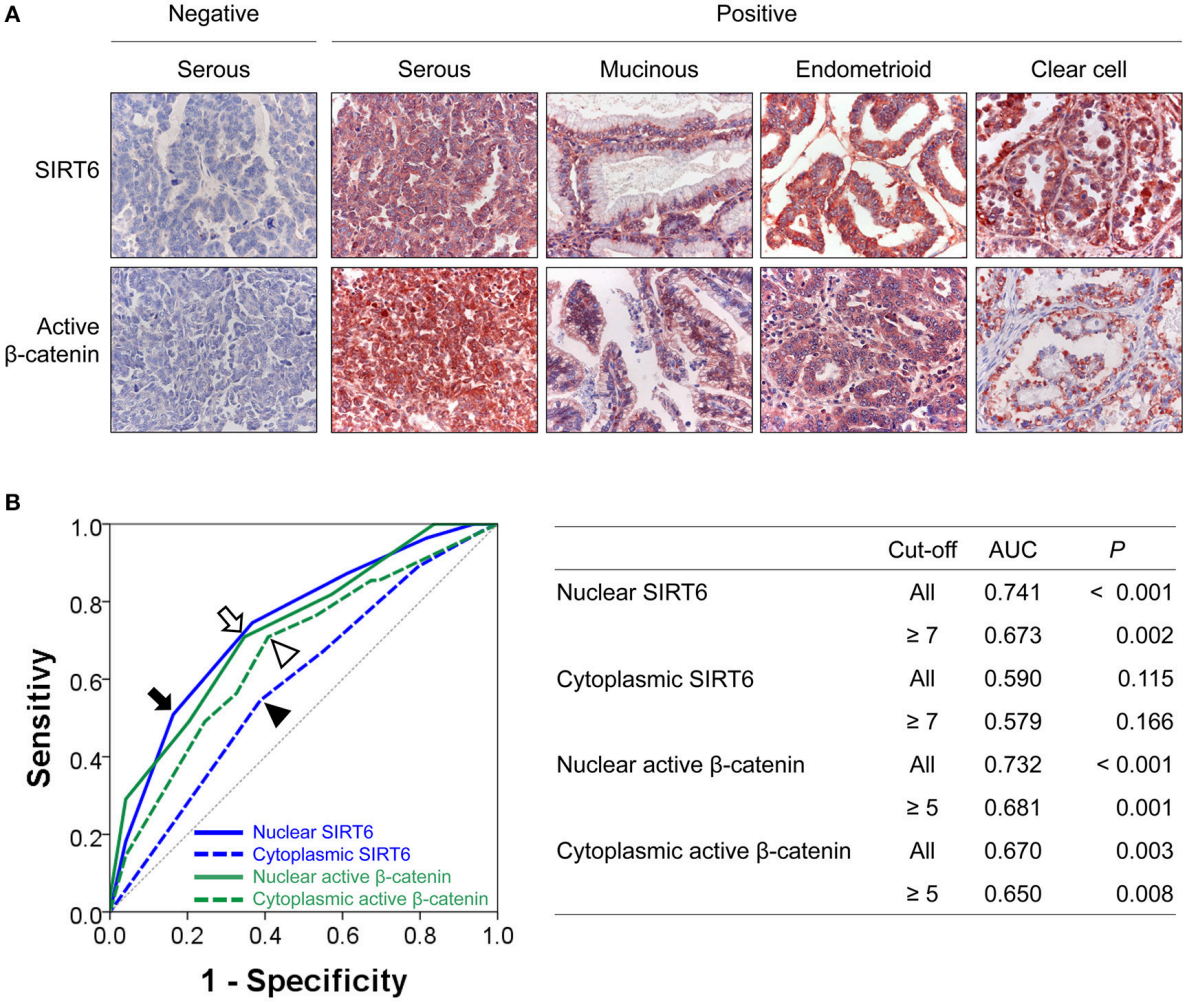
Immunohistochemical expressions of SIRT6 and active β-catenin, and statistical analysis in ovarian carcinomas. **(A)** Immunohistochemical expressions of SIRT6 and active β-catenin are seen in both the cytoplasm and nuclei of tumor cells. **(B)** Receiver operator characteristic curve analysis to determine the cut-off points for the immunohistochemical staining for SIRT6 and active β-catenin. The cut-off points were determined at the highest area under the curve (AUC) value. The cut-off points for nuclear expression of SIRT6 (arrow), cytoplasmic expression of SIRT6 (arrow head), nuclear expression of active β-catenin (empty head), and cytoplasmic expression of active β-catenin (empty arrow head) were seven, seven, five, and five, respectively. The table shows AUC and *P* values at cut-off points for the nuclear and cytoplasmic expressions of SIRT6 and active β-catenin.

In human ovarian carcinomas, immunohistochemical expression of SIRT6 and β-catenin was observed in both the cytoplasm and the nuclei of tumor cells (Figure 1A). The nuclear and cytoplasmic expression patterns of SIRT6 and active β-catenin were classified into negative or positive based on the highest predictive points to estimate survival of ovarian carcinoma patients by receiver operating characteristic curve analysis. The cut-off points for the expressions of nuclear SIRT6 (Nu-SIRT6), cytoplasmic SIRT6 (Cy-SIRT6), nuclear active β-catenin (Nu-Aβ-catenin), and cytoplasmic active β-catenin (Cy-Aβ-catenin) were seven, seven, five, and five, respectively (Figure 1B). The expression of Nu-SIRT6 and Cy-SIRT6 were considered positive when the scores were ≥7, and the expression of Nu-Aβ-catenin and Cy-Aβ-catenin were considered positive when the scores were ≥5. With these cut-off values, Nu-SIRT6-positivity was significantly associated with elevated preoperative serum level of CA125, higher tumor stage, presence of ascites, bilaterality of the tumor, higher histologic grade, platinum resistance, and the expressions of Cy-SIRT6, Nu-Aβ-catenin, and Cy-Aβ-catenin (Table 2). The expression of Cy-SIRT6 was significantly associated with serum level of CA125, tumor stage, bilaterality of the tumor, histologic grade, platinum resistance, and the expressions of Nu-Aβ-catenin, and Cy-Aβ-catenin (Table 2). Nu-Aβ-catenin-positivity was significantly associated with elevated serum level of CA125, higher tumor stage, bilaterality of the tumor, higher histologic grade, histologic type, platinum resistance, and Cy-Aβ-catenin expression (Table 2). The expression of Cy-Aβ-catenin was significantly associated with serum level of CA125, tumor stage, bilaterality of the tumor, histologic grade, and histologic type (Table 2).

**Table 2:** Clinicopathological variables and expression of SIRT6 and active β-catenin in 104 ovarian carcinomas.

**Characteristics**		**No**.	**Nu-SIRT6**		**Cy-SIRT6**		**Nu-A**β**-catenin**		**Cy-A**β**-catenin**	
			**Positive**	***P***	**Positive**	***P***	**Positive**	***P***	**Positive**	***P***
Age, y	< 60	71	21 (30%)	0.113	33 (46%)	0.849	34 (48%)	0.074	38 (54%)	0.333
	≥ 60	33	15 (45%)		16 (48%)		22 (67%)		21 (64%)	
CA125	Normal	18	2 (11%)	0.013	3 (17%)	0.003	6 (33%)	0.032	6 (33%)	0.013
	Elevated	75	32 (43%)		42 (56%)		46 (61%)		49 (65%)	
Stage	I & II	52	10 (19%)	< 0.001	19 (37%)	0.031	22 (42%)	0.018	24 (46%)	0.029
	III & IV	52	26 (50%)		30 (58%)		34 (65%)		35 (67%)	
Tumor size, cm	≤ 10	68	24 (35%)	0.842	33 (49%)	0.691	40 (59%)	0.162	43 (63%)	0.066
	> 10	36	12 (33%)		16 (44%)		16 (44%)		16 (44%)	
LN metastasis	Absence	84	27 (32%)	0.277	39 (46%)	0.774	43 (51%)	0.266	44 (52%)	0.067
	Presence	20	9 (45%)		10 (50%)		13 (65%)		15 (75%)	
Ascites	Absence	71	19 (27%)	0.014	30 (42%)	0.145	34 (48%)	0.074	39 (55%)	0.587
	Presence	33	17 (52%)		19 (58%)		22 (67%)		20 (61%)	
Bilaterality	Unilateral	59	13 (22%)	0.002	22 (37%)	0.022	24 (41%)	0.002	26 (44%)	0.003
	Bilateral	45	23 (51%)		27 (60%)		32 (71%)		33 (73%)	
Histologic grade	Low (1)	27	4 (15%)	0.012	6 (22%)	0.003	8 (30%)	0.003	8 (30%)	< 0.001
	High (2 and 3)	77	32 (42%)		43 (56%)		48 (62%)		51 (66%)	
Histologic type	Serous	75	29 (39%)	0.669	37 (49%)	0.353	51 (68%)	< 0.001	53 (71%)	< 0.001
	Mucinous	20	5 (25%)		6 (30%)		2 (10%)		1 (5%)	
	Endometrioid	5	1 (20%)		3 (60%)		1 (20%)		2 (40%)	
	Clear cell	3	1 (33%)		2 (67%)		2 (67%)		2 (67%)	
	Malignant Brenner	1	0 (0%)		1 (100%)		0 (0%)		1 (100%)	
Platinum-resistance	Absence	62	18 (29%)	0.001	25 (40%)	0.021	29 (47%)	0.009	34 (55%)	0.110
	Presence	20	14 (70%)		14 (70%)		16 (80%)		15 (75%)	
Cy-Aβ-catenin	Negative	45	9 (20%)	0.006	13 (29%)	0.001	6 (13%)	< 0.001		
	Positive	59	27 (46%)		36 (61%)		50 (85%)			
Nu-Aβ-catenin	Negative	48	9 (19%)	0.002	16 (33%)	0.009				
	Positive	56	27 (48%)		33 (59%)					
Cy-SIRT6	Negative	55	8 (15%)	< 0.001						
	Positive	49	28 (57%)							

*Nu-SIRT6, nuclear expression of SIRT6; Cy-SIRT6, cytoplasmic expression of SIRT6; Nu-Aβ-catenin, nuclear expression of active β-catenin; Cy-Aβ-catenin, cytoplasmic expression of active β-catenin, LN, lymph node*.

### The expressions of sirt6 and active β-catenin are significantly associated with shorter survival of ovarian carcinoma patients by univariate analysis

**Figure 2:**
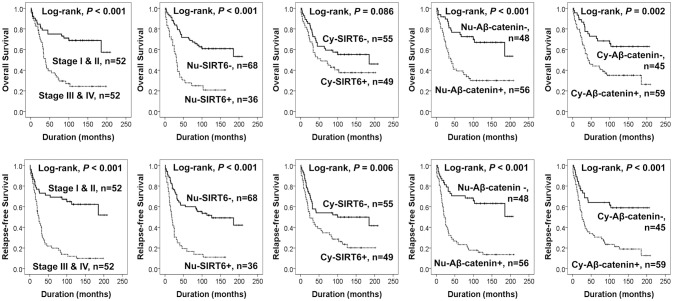
Kaplan-Meier survival analysis in 104 ovarian carcinomas. Overall survival and relapse-free survival according to tumor stage and immunohistochemical expression of SIRT6 and active β-catenin in tumor cells. Nu-SIRT6, nuclear expression of SIRT6; Cy-SIRT6, cytoplasmic expression of SIRT6; Nu-Aβ-catenin, nuclear expression of active β-catenin; Cy-Aβ-catenin, cytoplasmic expression of active β-catenin.

In 104 ovarian carcinomas, the factors significantly associated with both OS and RFS in univariate analysis were age of patients, tumor stage, presence of ascites, serum level of CA125, histologic grade, and the expression of Nu-SIRT6, Nu-Aβ-catenin, and Cy-Aβ-catenin (Table 3) (Figure 2). Nu-SIRT6 positivity predicted a 3.280-fold (95% confidential interval (95% CI) 1.908-5.638) greater risk of death and a 3.252-fold (95% CI; 1.977–5.349) greater risk of relapse or death (Table 3). Nu-Aβ-catenin positivity also predicted a 2.940-fold (95% CI; 1.638–5.278) greater risk of death and a 3.761-fold (95% CI; 2.172–6.512) greater risk of relapse or death of ovarian carcinoma patients (Table 3). The expression of Cy-SIRT6 was significantly associated with RFS but not OS (Table 3). The Kaplan-Meier survival curves of tumor stage and expression of Nu-SIRT6, Cy-SIRT6, Nu-Aβ-catenin, and Cy-Aβ-catenin for OS and RFS are presented in Figure 2.

**Table 3:** Univariate Cox regression analyses for overall survival and relapse-free survival in 104 ovarian carcinomas and 63 high-grade serous carcinomas.

**Characteristics**	**No**.	**OS**		**RFS**	
		**HR (95% CI)**	***P***	**HR (95% CI)**	***P***
**OVERALL OVARIAN CARCINOMAS (*****n*** = **104)**					
Age, y, ≥ 60 (vs. < 60)	33/104	2.647 (1.554–4.511)	< 0.001	2.248 (1.375–3.673)	0.001
Stage, III & IV (vs. I & II)	52/104	3.232 (1.809–5.774)	< 0.001	3.898 (2.269–6.695)	< 0.001
Tumor size, cm, > 10 (vs. ≤ 10)	36/104	0.502 (0.270–0.936)	0.030	0.588 (0.342–1.012)	0.055
LN metastasis, presence (*vs*. absence)	20/104	1.382 (0.739–2.583)	0.311	1.738 (0.996–3.035)	0.052
Ascites, presence (*vs*. absence)	33/104	1.902 (1.106–3.272)	0.020	1.825 (1.111–2.998)	0.018
Bilaterality, bilateral (vs. unilateral)	45/104	1.612 (0.947–2.745)	0.079	2.050 (1.254–3.350)	0.004
CA125, elevated (vs. normal)	75/93	5.013 (1.554–16.167)	0.007	4.873 (1.760–13.490)	0.002
Histologic grade, high (vs. low)	77/104	3.626 (1.548–8.493)	0.003	3.605 (1.715–7.582)	< 0.001
Nu-SIRT6, positive (vs. negative)	36/104	3.280 (1.908–5.638)	< 0.001	3.252 (1.977–5.349)	< 0.001
Cy-SIRT6, positive (vs. negative)	49/104	1.583 (0.930–2.696)	0.091	1.952 (1.194–3.190)	0.008
Nu-Aβ-catenin, positive (vs. negative)	56/104	2.940 (1.638–5.278)	< 0.001	3.761 (2.172–6.512)	< 0.001
Cy-Aβ-catenin, positive (vs. negative)	59/104	2.417 (1.348–4.334)	0.003	2.889 (1.673–4.987)	< 0.001
**HIGH-GRADE SEROUS CARCINOMAS (n** = **63)**					
Age, y, ≥ 60 (vs. < 60)	27/63	2.043 (1.125–3.710)	0.019	1.648 (0.954–2.848)	0.073
Stage, III & IV (vs. I & II)	38/63	1.953 (1.009–3.782)	0.047	2.838 (1.502–5.364)	0.001
Tumor size, cm, > 10 (vs. ≤ 10)	15/63	0.773 (0.371–1.612)	0.492	0.811 (0.424–1.551)	0.526
LN metastasis, presence (vs. absence)	15/63	1.401 (0.714–2.749)	0.327	1.804 (0.975–3.337)	0.060
Ascites, presence (vs. absence)	26/63	1.349 (0.740–2.459)	0.329	1.357 (0.782–2.355)	0.277
Bilaterality, bilateral (vs. unilateral)	36/63	1.339 (0.719–2.493)	0.358	1.593 (0.893–2.841)	0.115
CA125, elevated (vs. normal)	53/58	2.454 (0.590–10.218)	0.217	1.614 (0.500–5.211)	0.424
Nu-SIRT6, positive (vs. negative)	26/63	2.433 (1.323–4.475)	0.004	2.211 (1.261–3.878)	0.006
Cy-SIRT6, positive (vs. negative)	34/63	1.126 (0.621–2.041)	0.696	1.216 (0.702–2.105)	0.485
Nu-Aβ-catenin, positive (vs. negative)	45/63	3.284 (1.515–7.118)	0.003	3.600 (1.781–7.275)	< 0.001
Cy-Aβ-catenin, positive (vs. negative)	46/63	1.682 (0.830–3.411)	0.149	1.672 (0.874–3.198)	0.120

*Nu-SIRT6, nuclear expression of SIRT6; Cy-SIRT6, cytoplasmic expression of SIRT6; Nu-Aβ-catenin, nuclear expression of active β-catenin; Cy-Aβ-catenin, cytoplasmic expression of active β-catenin; LN, lymph node*.

**Figure 3:**
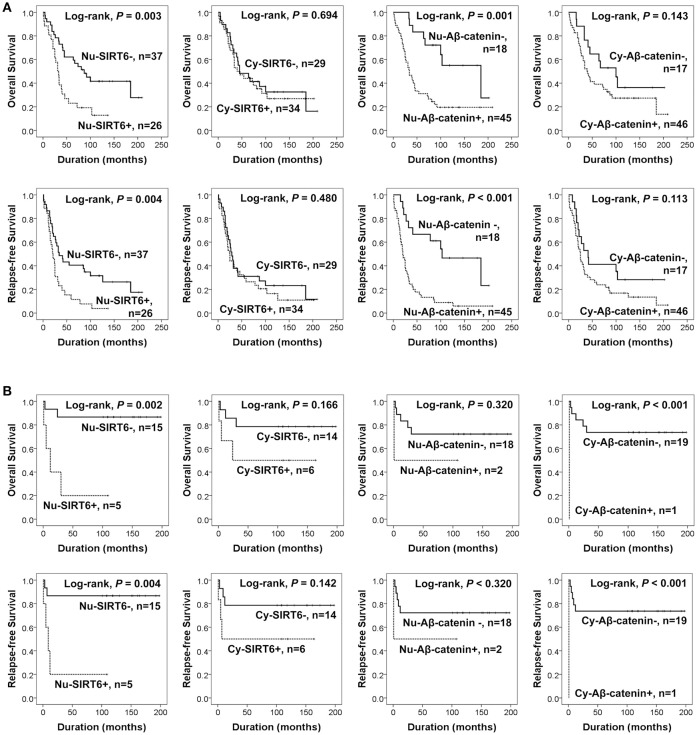
Survival analysis according to the expression of SIRT6 and active β-catenin in the subgroups of high-grade serous carcinomas and mucinous carcinomas of the ovary. **(A)** Kaplan-Meier survival analysis for overall survival and relapse-free survival in the 75 high-grade serous carcinomas. **(B)** Kaplan-Meier survival analysis for overall survival and relapse-free survival in 20 mucinous carcinomas. Nu-SIRT6, nuclear expression of SIRT6; Cy-SIRT6, cytoplasmic expression of SIRT6; Nu-Aβ-catenin, nuclear expression of active β-catenin; Cy-Aβ-catenin, cytoplasmic expression of active β-catenin.

Additionally, we performed further analysis of survival in high-grade serous carcinomas and mucinous carcinomas. The factors significantly associated with OS or RFS by univariate analysis in 63 high-grade serous carcinomas were age of patients, tumor stage, Nu-SIRT6 expression, and Nu-Aβ-catenin expression (Table 3). The Kaplan-Meier survival analysis of high-grade serous carcinomas and mucinous carcinomas are presented in Figure 3. In 20 mucinous carcinomas, Nu-SIRT6 expression and Cy-Aβ-catenin expression were significantly associated with both OS and RFS (Figure 3B).

### Nuclear expressions of SIRT6 and active β-catenin are significantly associated with shorter survival of ovarian carcinoma patients by multivariate analysis

Multivariate analysis was performed with the factors significantly associated with OS or RFS. However, the serum level of CA125 was not included in the multivariate analysis because it was not evaluated in eleven patients. In 104 ovarian carcinomas, the variables included in the multivariate analysis were age, tumor stage, tumor size, presence of ascites, bilaterality of the tumor, histologic grade, Nu-SIRT6 expression, Cy-SIRT6 expression, Nu-Aβ-catenin expression, and Cy-Aβ-catenin expression. The factors significantly associated with both OS or RFS in multivariate analysis were age of patients, tumor stage, bilaterality of the tumor, histologic grade, Nu-SIRT6 expression, and Nu-Aβ-catenin expression (Table 4). The patients with Nu-SIRT6-positive tumors had a 2.087-fold (95% CI' 1.198–3.651) greater risk of shorter OS compared with the patients with Nu-SIRT6-negative tumor. The patients with Nu-Aβ-catenin-positive tumors had a 2.158-fold (95% CI; 1.176–3.960) greater risk of shorter OS and a 3.131-fold (95% CI; 1.741–5.633) greater risk of shorter RFS compared with the patients with Nu-Aβ-catenin-negative tumors (Table 4).

**Table 4:** Multivariate Cox regression analyses for overall survival and relapse-free survival in 104 ovarian carcinomas and 63 high-grade serous carcinomas.

**Characteristics**	**OS**		**RFS**	
	**HR (95% CI)**	***P***	**HR (95% CI)**	***P***
**OVERALL OVARIAN CARCINOMAS (*****n*** = **104)**^a^				
Age, y, ≥ 60 (vs. < 60)	2.049 (1.195–3.514)	0.009	1.687 (1.029–2.767)	0.038
Stage, III & IV (vs. I & II)			4.167 (2.096–8.284)	< 0.001
Bilaterality, bilateral (vs. unilateral)			0.506 (0.266–0.963)	0.038
Histologic grade, high (vs. low)	2.408 (1.013–5.723)	0.047	2.682 (1.262–5.699)	0.010
Nu-SIRT6, positive (vs. negative)	2.087 (1.193–3.651)	0.010	
Nu-Aβ-catenin, positive (vs. negative)	2.158 (1.176–3.960)	0.013	3.131 (1.741–5.633)	< 0.001
**HIGH-GRADE SEROUS CARCINOMAS (*****n*** = **63)**^b^				
Age, y, ≥ 60 (vs. < 60)	2.185 (1.195–3.996)	0.011	
Stage, III & IV (vs. I & II)			2.418 (1.271–4.601)	0.007
Nu-SIRT6, positive (vs. negative)	1.905 (1.030–3.524)	0.040	
Nu-Aβ-catenin, positive (vs. negative)	3.078 (1.030–3.524)	0.005	3.160 (1.553–6.433)	0.002

*Nu-SIRT6, nuclear expression of SIRT6; Nu-Aβ-catenin, nuclear expression of active β-catenin*.

a *Variables included in multivariate analysis were age, tumor stage, tumor size, presence of ascites, bilaterality of the tumor, histologic grade, nuclear expression of SIRT6, cytoplasmic expression of SIRT6, nuclear expression of active β-catenin, and cytoplasmic expression of active β-catenin*.

b *Variables included in multivariate analysis were age, tumor stage, nuclear expression of SIRT6, and nuclear expression of active β-catenin*.

In 63 high-grade serous carcinomas, the factors included in the multivariate analysis were age, tumor stage, Nu-SIRT6 expression, and Nu-Aβ-catenin expression. Older age of patients was associated with shorter OS and higher tumor stage was associated with shorter RFS (Table 4). Nu-SIRT6 positivity predicted a 1.905-fold (95% CI; 1.030–3.524) greater risk of death of high-grade serous carcinoma patients. Nu-Aβ-catenin positivity predicted a 3.078-fold (95% CI; 1.030–3.524) greater risk of shorter OS and a 3.160-fold (95% CI; 1.553–6.433) greater risk of shorter RFS (Table 4). Because of the limited number of cases, we did not perform multivariate analysis of mucinous carcinomas.

### SIRT6 expression is associated with invasiveness of ovarian cancer cells

**Figure 4:**
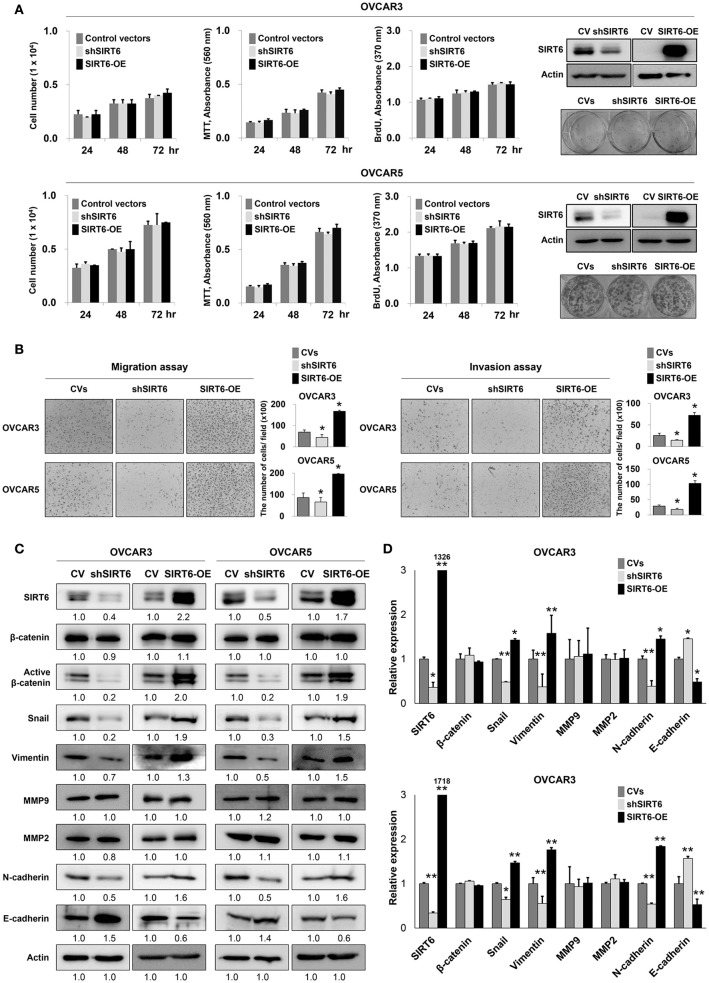
The effect of SIRT6 on the proliferation and invasiveness of ovarian cancer cells. **(A)** Counting the number of cells, MTT, BrdU incorporation, and colony forming assays were performed to evaluate the proliferation of OVCAR3 and OVCAR5 ovarian cancer cells after knock-down of SIRT6 with shRNA for SIRT6 or overexpression of SIRT6 with wild-type SIRT6. Western blotting was performed to show knock-down or overexpression of SIRT6. **(B)** Migration and invasion assays were performed to evaluate the invasiveness of OVCAR3 and OVCAR5 cells after knock-down of SIRT6 or overexpression of SIRT6. The number of cells which migrated or invaded the chambers were counted in five microscopic fields per well at one-hundred magnification. **(C)** Western blotting was performed for SIRT6, β-catenin, active β-catenin, snail, vimentin, MMP9, MMP2, N-cadherin, E-cadherin, and actin after knock-down of SIRT6 or overexpression of SIRT6 in OVCAR3 and OVCAR5 cells. The western bands were quantified using ImageJ software and values are indicated below the bands. **(D)** Quantitative reverse-transcription polymerase chain reaction was performed for SIRT6, β-catenin, snail, vimentin, MMP9, MMP2, N-cadherin, and E-cadherin after knock-down of SIRT6 or overexpression of SIRT6 in OVCAR3 and OVCAR5 cells. CV, control vector; OE, overexpression; *, vs. control; *P* < 0.05.

In human ovarian carcinomas, the expression of SIRT6 and active β-catenin were associated with advanced clinicopathological factors and shorter survival of patients. Therefore, we further evaluated the effects of SIRT6 expression on the proliferation and invasiveness of ovarian cancer cells. In OVCAR3 and OVCAR5 ovarian cancer cells, knock-down of SIRT6 with shRNA for SIRT6 or overexpression of SIRT6 did not influence the proliferation of cells in the counting of cells, MTT, BrdU incorporation, and colony-forming assays (Figure 4A). However, knock-down of SIRT6 significantly inhibited migration and invasive activity of both OVCAR3 and OVCAR5 ovarian cancer cells. The migration activity of OVCAR3 and OVCAR5 cells decreased to 63% (*P* = 0.049) and 76% (*P* = 0.049) with overexpression of SIRT6 compared with cells transfected control vector, respectively. The migration activity of OVCAR3 and OVCAR5 cells increased to 242% (*P* = 0.006) and 226% (*P* = 0.019) with overexpression of SIRT6 compared with cells transfected control vector, respectively. The invasion activity of OVCAR3 and OVCAR5 cells were decreased to 57% (*P* = 0.009) and 62% (*P* = 0.027) with overexpression of SIRT6 compared with the cells transfected control vector, respectively. The invasion activity of OVCAR3 and OVCAR5 cells were increased to 283% (*P* = 0.015) and 358% (*P* = 0.008) with overexpression of SIRT6 compared with the cells transfected control vector, respectively. Transfection with SIRT6 significantly increased migration and invasive activity of both OVCAR3 and OVCAR5 cells (Figure 4B). Based on the results of migration and invasion assays, we evaluated the expression of mRNAs and proteins involved in invasiveness of cancer cells via western blot. Knock-down of SIRT6 decreased the expression of mRNAs and proteins of snail, vimentin, and N-cadherin, and increased the expression of mRNAs and proteins of E-cadherin in both OVCAR3 and OVCAR5 cells. Conversely, overexpression of SIRT6 increased the expression of mRNAs and proteins of snail, vimentin, and N-cadherin, and decreased the expression of mRNAs and proteins of E-cadherin in both OVCAR3 and OVCAR5 cells (Figures 4C,D). Although the expression of mRNAs and proteins of β-catenin was not changed with SIRT6 expression level, the expression of active β-catenin protein was decreased with knock-down of SIRT6 and increased with overexpression of SIRT6 (Figures 4C,D). The expression of mRNAs and proteins of MMP2 and MMP9 were unaffected by either knock-down or overexpression of SIRT6.

### SIRT6 and β-catenin are involved in invasiveness of ovarian cancer cells

**Figure 5:**
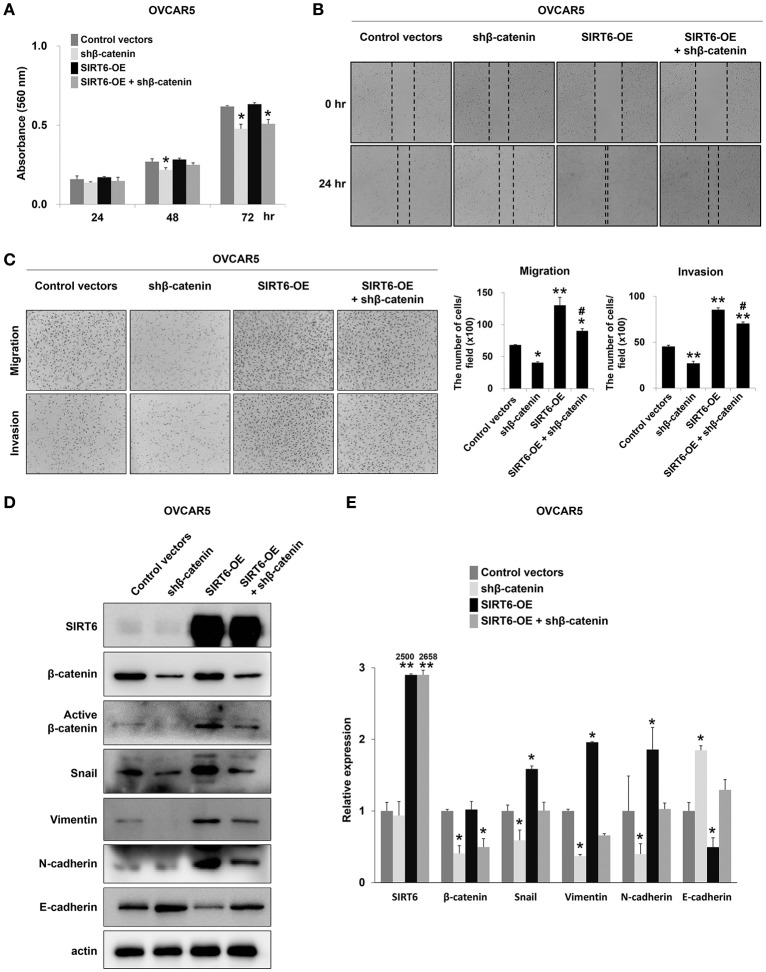
Knock-down of β-catenin attenuates the effect of SIRT overexpression in invasion activity of ovarian cancer cells. **(A)** The MTT assay was performed after overexpression of SIRT6 with or without knock-down of β-catenin. **(B,C)** SIRT6 was overexpressed via a plasmid containing wild-type SIRT6 and β-catenin was knocked-down with shRNA for β-catenin in OVCAR5 ovarian cancer cells. Wound healing assays **(B)**, and migration and invasion assays **(C)** were performed after overexpression of SIRT6 with or without knock-down of β-catenin in OVCAR5 cells. The number of cells which migrated or invaded the chambers were counted in five microscopic fields per well at one-hundred magnification. **(D)** Western blotting was performed for SIRT6, β-catenin, active β-catenin, snail, vimentin, N-cadherin, E-cadherin, and actin after overexpression of SIRT6 with or without knock-down of β-catenin in OVCAR5 cells. **(E)** The mRNA expression patterns for SIRT6, β-catenin, snail, vimentin, N-cadherin, and E-cadherin were analyzed *via* quantitative reverse-transcription polymerase chain reaction after overexpression of SIRT6 with or without knock-down of β-catenin in OVCAR5 cells. CV, control vector; OE, overexpression; the *P* values were calculated by one-way ANOVA with LSD test; *, vs. control; *P* < 0.05, **, vs. control; *P* < 0.001, ^#^, vs. SIRT6 overexpression; *P* < 0.05.

As shown in Figure 4, and consistent with our previous report that SIRT6 expression is associated with active β-catenin expression in breast cancer cells (22), SIRT6 expression was associated with increased expression of active β-catenin and invasiveness of ovarian cancer cells. Therefore, to evaluate the relationship between SIRT6 and β-catenin in the invasiveness of ovarian cancers, we co-transfected a SIRT6 overexpression vector and shRNA for β-catenin. As shown in Figure 4, the overexpression of SIRT6 did not influence the proliferation of ovarian cancer cells. However, knock-down of β-catenin inhibited both proliferation and invasiveness of OVCAR5 cells (Figures 5A–C). Knock-down of β-catenin decreased the expression of protein and mRNA of snail, vimentin, and N-cadherin, and increased expression of protein and mRNA of E-cadherin (Figures 5D,E). Moreover, increased invasiveness of OVCAR5 cells with SIRT6 overexpression was attenuated by a knock-down of β-catenin (Figures 5B,C). In addition, increased expression of protein and mRNA of snail, vimentin, and N-cadherin resulting from overexpression of SIRT6, were suppressed with knock-down of β-catenin, and SIRT6-mediated suppression of E-cadherin expression was increased with knock-down of β-catenin (Figures 5D,E).

## Discussion

In this study, we evaluated the expressions of SIRT6 and active β-catenin in human ovarian carcinomas and ovarian cancer cells. In human ovarian carcinomas, there was a significant correlation between the expression of SIRT6 and active β-catenin, and their expression patterns were significantly associated with advanced clinicopathologic factors of ovarian carcinomas such as elevated serum level of CA125, higher tumor stage, and higher histologic grade. Moreover, nuclear expression of SIRT6 and active β-catenin were independent indicators of poor prognosis in both the overall ovarian carcinomas and high-grade serous carcinomas. In addition, although the number of cases of mucinous carcinomas was limited, our results suggest that the expression patterns of SIRT6 and active β-catenin as possible prognostic indicators of mucinous carcinoma of the ovary. These findings suggest that the expression patterns of SIRT6 and active β-catenin are helpful for the prediction of survival of ovarian carcinoma patients. Consistent with our results, immunohistochemical expression of SIRT6 was higher in colon cancer tissue than adjacent normal tissue, and was associated with higher T stage, presence of lymph node metastasis, and higher histologic grade of colon cancers (24). In thyroid papillary carcinomas, higher expression of SIRT6 was associated with increased risk of lymph node metastasis (21). In addition, higher expression of SIRT6 was associated with shorter survival of gastric cancer patients (23) and non-small cell lung cancer patients (19). In breast cancers, both the nuclear and cytoplasmic expression of SIRT6 were associated with shorter survival of patients (22). In addition, when considering the role of nuclear β-catenin in the activation of T cell transcription factor to induce cellular proliferation (36), active β-catenin is expected to be expressed in advanced cancers. In agreement with our results that indicate Nu-Aβ-catenin expression to be an independent indicator of shorter survival of ovarian carcinomas, nuclear expression of β-catenin is an indicator of poor prognosis for various human cancers (37–39). Therefore, our results suggest SIRT6 and active β-catenin expression as indicators of poor prognosis for ovarian carcinomas. However, there are controversial reports regarding the prognostic significance of SIRT6 expression in human cancers. Immunohistochemical expression of SIRT6 was associated with favorable prognosis of breast cancer patients (29), and SIRT6 knockout was associated with shorter survival of hepatocellular carcinoma patients (12). In colon cancer, decreased expression of Nu-SIRT6 was associated with more frequent relapse in the subpopulation of patients with lymph node metastasis or higher levels of C-reactive protein (13). Therefore, further study is needed to clarify the prognostic role of SIRT6 expression in human cancers.

Our results, in addition to the prognostic significance of SIRT6 expression, there was a significant association between the nuclear/cytoplasmic expression of SIRT6 and the nuclear/cytoplasmic expression of active β-catenin in ovarian cancer tissue samples. Consistently, the expression of SIRT6 was associated with the expression of active β-catenin in ovarian cancer cells. These findings suggest that the SIRT6-β-catenin pathway might be involved in the progression of ovarian cancers. In addition, SIRT6-mediated invasiveness of ovarian cancer cells was associated with the expression of EMT signaling molecules such as E-cadherin, N-cadherin, snail, vimentin, and active β-catenin. Although MMP2/MMP9 expression was not influenced by the SIRT6 expression, SIRT6 overexpression decreased the expression of E-cadherin and increased the expression of active β-catenin, snail, N-cadherin, and vimentin. Therefore, our results suggest that SIRT6 expression is closely associated with invasiveness of ovarian carcinomas in conjunction with β-catenin expression. In agreement with our results, SIRT6 is involved in EMT of various human malignant tumors (22, 24, 40). In osteosarcoma cells, SIRT6-mediated invasiveness was associated with MMP9 signaling. Knock-down of SIRT6 significantly inhibited the invasiveness of SaOS2 and MG63 osteosarcoma cells by regulating the ERK1/2-MMP9 pathway (24). In pancreatic cancer cells, SIRT6-mediated increase of migration activity was mediated by Ca^2+^, and SIRT6 increased the expression of pro-inflammatory cytokines such as IL8 and TNF (40). In breast cancer cells, SIRT6 expression was associated with increased expression of MMP, cyclin D1, and NFκB, and was involved in nuclear localization of β-catenin (22). Therefore, when considering EMT as one of the molecular hallmarks of cancer progression (41, 42), the prognostic significance of SIRT6 and active β-catenin expression might be related with their roles in EMT of ovarian carcinomas. However, there is also a controversial report that SIRT6 inhibits EMT in a model of pulmonary fibrosis (3). Therefore, further study is needed to clarify the exact role of SIRT6 in cancer invasiveness.

Another aspect of our results is that SIRT6 expression was associated with active β-catenin expression. Despite the role of SIRT6 on the expression of active β-catenin, SIRT6 did not influence the proliferation of ovarian cancer cells. In contrast, β-catenin expression was involved in both proliferation and invasiveness of ovarian cancer cells. Moreover, SIRT6 mediated increase of invasiveness of ovarian cancer cells was attenuated by knock-down of β-catenin. Although the exact mechanism is not clear, these findings suggest that the role of SIRT6 on β-catenin signaling might be restricted to the invasiveness of ovarian cancer cells. Similarly, SIRT6 was involved in the invasiveness but not the proliferation of osteosarcoma cells (24). In breast cancers, SIRT6 increased both the proliferation and invasiveness of cancer cells (22). However, there were also controversial reports that SIRT6 inhibits the proliferation of pancreatic (14) and gastric cancer cells (15). Therefore, further study is needed to clarify the role(s) of SIRT6/β-catenin signaling in the progression of ovarian cancers.

As discussed previously, the role of SIRT6 in ovarian cancers has been limited to the invasiveness of cancer cells. However, despite the limited role of SIRT6 in ovarian cancer cells, SIRT6 showed a significant prognostic role in human ovarian carcinomas. These results raise the possibility of another role of SIRT6 in cancer progression, and that might be resistance to anti-cancer therapies in SIRT6-expressing ovarian cancers. In our ovarian carcinomas, platinum resistance was seen in 70% (14 of 20) of Nu-SIRT6-positive patients, but platinum resistance was only 30% (6 of 20) in Nu-SIRT6-negative patients. Consistently, SIRT6-mediated chemo-sensitization has been reported in various studies. SIRT6 increased resistance to paclitaxel and epirubicin in MCF7 breast cancer cells (28). In acute myeloid leukemia cells, inhibition of SIRT6 disrupts DNA damage repair mechanisms and increases sensitivity to daunorubicin and Ara-C (25). Depletion of SIRT6 sensitized pancreatic cancer cells to gemcitabine (26), and hepatocellular carcinoma cells to chemotherapeutic agents *via* downregulation of MDR1 expression (43). In addition, SIRT6-mediated chemoresistance might be related with its role in the regulation of EMT because the EMT molecules snail and E-cadherin, whose expression is affected by SIRT6, are involved in chemoresistance. Snail regulates chemoresistance of breast cancer cells (44) and E-cadherin expression was associated with sensitivity to EGFR kinase inhibitors (45). Consistently, E-cadherin-positivity was associated with the longest survival, and snail-positivity was associated with shorter survival of breast cancer patients (46).

Another mechanism for how SIRT6 induces chemoresistance might be related with the role of SIRT6 in DNA damage repair by activating PARP1 (6). In some contexts, such as treatment on human cancers with genotoxic agents, intact DNA damage repair pathways confer resistance (47). The favorable prognosis of cancer patients with a defect in BRCA1/2 (34) and the poor prognosis with BRCA1/2-expressing cancers have been reported in various cancers (30, 48–50). Therefore, based on the role of DNA damage response signaling in chemoresistance, PARP inhibitors are approved for the treatment of advanced ovarian cancers (51). In addition, when we evaluated the association between the expression of BRCA1 and SIRT6/active β-catenin based on our previous report (30), there was a significant correlation between their expression patterns (Supplementary Table 1). Therefore, the role of SIRT6 in DNA damage repair might endow cells with resistance to therapy. However, there are also controversial reports on the role of SIRT6 in chemoresistance. In hepatocellular carcinomas, SIRT6 increased sensitivity to chemotherapeutic agent-mediated apoptosis of HepG2 cells (12). In addition, loss of SIRT6 was associated with resistance to trastuzumab in breast cancer cells (29). Therefore, further study is needed to clarify the role of SIRT6 in cancer progression and the treatment of cancer. Especially, although we did not perform such a test, a pre-clinical *in vivo* experiment might be helpful in understanding the role of SIRT6 in cancer progression.

In conclusion, although there are controversial reports on the role of SIRT6 in human malignant tumors, this is the first study to demonstrate that SIRT6 is involved in the invasiveness of ovarian cancers in a mechanism involving the expression of active β-catenin and EMT signaling. Moreover, our results indicate nuclear expression patterns of SIRT6 and active β-catenin to be important prognostic indicators of ovarian cancers, especially in high-grade serous carcinomas. Therefore, our results suggest that SIRT6 is involved in the progression of ovarian carcinomas and that inhibition of SIRT6 might be a new therapeutic stratagem for the treatment of ovarian cancers, especially in the cancers with high expression of SIRT6.

## Author contributions

JB, SN, KK, S-HP, HP, B-HP, SH, HL, MC, WM, DC, and KJ participated in the study design. JB, SN, KK, S-HP, UH, HP, DC, and KJ performed experiment. JB, SN, KK, UH, HP, HL, MC, WM, DC, and KJ were involved in data collection and data interpretation. JB, SN, KK, HP, DC, and KJ participated in the statistical analyses. JB, SN, KK, S-HP, UH, HP, B-HP, SH, HL, MC, WM, DC, and KJ wrote the manuscript. All authors read and approved the final manuscript.

### Conflict of interest statement

The authors declare that the research was conducted in the absence of any commercial or financial relationships that could be construed as a potential conflict of interest.

## References

[1] Mostoslavsky R Chua KF Lombard DB Pang WW Fischer MR Gellon L. Genomic instability and aging-like phenotype in the absence of mammalian SIRT6. Cell (2006) 124:315–29. 10.1016/j.cell.2005.11.044 16439206

[2] Kim HS Xiao C Wang RH Lahusen T Xu X Vassilopoulos A. Hepatic-specific disruption of SIRT6 in mice results in fatty liver formation due to enhanced glycolysis and triglyceride synthesis. Cell Metab. (2010) 12:224–36. 10.1016/j.cmet.2010.06.009 20816089 PMC2935915

[3] Tian K Chen P Liu Z Si S Zhang Q Mou Y. Sirtuin 6 inhibits epithelial to mesenchymal transition during idiopathic pulmonary fibrosis via inactivating TGF-beta1/Smad3 signaling. Oncotarget (2017) 8:61011–24. 10.18632/oncotarget.17723 28977842 PMC5617402

[4] Kanfi Y Naiman S Amir G Peshti V Zinman G Nahum L. The sirtuin SIRT6 regulates lifespan in male mice. Nature (2012) 483:218–21. 10.1038/nature10815 22367546

[5] Etchegaray JP Zhong L Mostoslavsky R. The histone deacetylase SIRT6: at the crossroads between epigenetics, metabolism and disease. Curr Top Med Chem. (2013) 13:2991–3000. 10.2174/15680266113136660213 24171769

[6] Mao Z Hine C Tian X Van Meter M Au M Vaidya A. SIRT6 promotes DNA repair under stress by activating PARP1. Science (2011) 332:1443–6. 10.1126/science.1202723 21680843 PMC5472447

[7] Xiong X Sun X Wang Q Qian X Zhang Y Pan X. SIRT6 protects against palmitate-induced pancreatic beta-cell dysfunction and apoptosis. J Endocrinol. (2016) 231:159–65. 10.1530/JOE-16-0317 27601447 PMC5365398

[8] Song MY Wang J Ka SO Bae EJ Park BH. Insulin secretion impairment in Sirt6 knockout pancreatic beta cells is mediated by suppression of the FoxO1-Pdx1-Glut2 pathway. Sci Rep. (2016) 6:30321. 10.1038/srep30321 27457971 PMC4960548

[9] Kaluski S Portillo M Besnard A Stein D Einav M Zhong L. Neuroprotective functions for the histone deacetylase SIRT6. Cell Rep. (2017) 18:3052–62. 10.1016/j.celrep.2017.03.008 28355558 PMC5389893

[10] Zhang S Jiang S Wang H Di W Deng C Jin Z. SIRT6 protects against hepatic ischemia/reperfusion injury by inhibiting apoptosis and autophagy related cell death. Free Radic Biol Med. (2018) 115:18–30. 10.1016/j.freeradbiomed.2017.11.005 29129519

[11] Samant SA Kanwal A Pillai VB Bao R Gupta MP. The histone deacetylase SIRT6 blocks myostatin expression and development of muscle atrophy. Sci Rep. (2017) 7:11877. 10.1038/s41598-017-10838-5 28928419 PMC5605688

[12] Marquardt JU Fischer K Baus K Kashyap A Ma S Krupp M. Sirtuin-6-dependent genetic and epigenetic alterations are associated with poor clinical outcome in hepatocellular carcinoma patients. Hepatology (2013) 58:1054–64. 10.1002/hep.26413 23526469 PMC3759627

[13] Sebastian C Zwaans BM Silberman DM Gymrek M Goren A Zhong L. The histone deacetylase SIRT6 is a tumor suppressor that controls cancer metabolism. Cell (2012) 151:1185–99. 10.1016/j.cell.2012.10.047 23217706 PMC3526953

[14] Kugel S Sebastian C Fitamant J Ross KN Saha SK Jain E. SIRT6 suppresses pancreatic cancer through control of Lin28b. Cell (2016) 165:1401–15. 10.1016/j.cell.2016.04.033 27180906 PMC4892983

[15] Zhou J Wu A Yu X Zhu J Dai H. SIRT6 inhibits growth of gastric cancer by inhibiting JAK2/STAT3 pathway. Oncol Rep. (2017) 38:1059–66. 10.3892/or.2017.5753 28656307

[16] Lee N Ryu HG Kwon JH Kim DK Kim SR Wang HJ. SIRT6 depletion suppresses tumor growth by promoting cellular senescence induced by DNA Damage in HCC. PLoS ONE (2016) 11:e0165835. 10.1371/journal.pone.0165835 27824900 PMC5100879

[17] Garcia-Peterson LM Ndiaye MA Singh CK Chhabra G Huang W Ahmad N. SIRT6 histone deacetylase functions as a potential oncogene in human melanoma. Genes Cancer (2017) 8:701–12. 10.18632/genesandcancer.153 29234488 PMC5724804

[18] Huang N Liu Z Zhu J Cui Z Li Y Yu Y. Sirtuin 6 plays an oncogenic role and induces cell autophagy in esophageal cancer cells. Tumour Biol. (2017) 39:1010428317708532. 10.1177/1010428317708532 28653878

[19] Bai L Lin G Sun L Liu Y Huang X Cao C. Upregulation of SIRT6 predicts poor prognosis and promotes metastasis of non-small cell lung cancer via the ERK1/2/MMP9 pathway. Oncotarget (2016) 7:40377–86. 10.18632/oncotarget.9750 27777384 PMC5130014

[20] Lin H Hao Y Zhao Z Tong Y. Sirtuin 6 contributes to migration and invasion of osteosarcoma cells via the ERK1/2/MMP9 pathway. FEBS Open Bio (2017) 7:1291–301. 10.1002/2211-5463.12265 28904859 PMC5586348

[21] Qu N Hu JQ Liu L Zhang TT Sun GH Shi RL SIRT6 is upregulated and associated with cancer aggressiveness in papillary thyroid cancer via BRAF/ERK/Mcl-1 pathway. Int J Oncol. (2017) 50:1683–92. 10.3892/ijo.2017.3951 28393212

[22] Bae JS Park SH Jamiyandorj U Kim KM Noh SJ Kim JR. CK2alpha/CSNK2A1 phosphorylates SIRT6 and is involved in the progression of breast carcinoma and predicts shorter survival of diagnosed patients. Am J Pathol. (2016) 186:3297–315. 10.1016/j.ajpath.2016.08.007 27746184

[23] Shen X Li P Xu Y Chen X Sun H Zhao Y. Association of sirtuins with clinicopathological parameters and overall survival in gastric cancer. Oncotarget (2017) 8:74359–70. 10.18632/oncotarget.20799 29088792 PMC5650347

[24] Geng CH Zhang CL Zhang JY Gao P He M Li YL. Overexpression of Sirt6 is a novel biomarker of malignant human colon carcinoma. J Cell Biochem. (2017) 119:3957–67. 10.1002/jcb.26539 29227545

[25] Cagnetta A Soncini D Orecchioni S Talarico G Minetto P Guolo F. Depletion of SIRT6 enzymatic activity increases acute myeloid leukemia cells' vulnerability to DNA-damaging agents. Haematologica (2018) 103:80–90. 10.3324/haematol.2017.176248 29025907 PMC5777193

[26] Damonte P Sociali G Parenti MD Soncini D Bauer I Boero S. SIRT6 inhibitors with salicylate-like structure show immunosuppressive and chemosensitizing effects. Bioorg Med Chem. (2017) 25:5849–58. 10.1016/j.bmc.2017.09.023 28958848

[27] Stewart BW Wild C International Agency for Research on Cancer, World Health Organization: World cancer report 2014. International Agency for Research on Cancer, Lyon, France (2014)

[28] Khongkow M Olmos Y Gong C Gomes AR Monteiro LJ Yague E. SIRT6 modulates paclitaxel and epirubicin resistance and survival in breast cancer. Carcinogenesis (2013) 34:1476–86. 10.1093/carcin/bgt098 23514751

[29] Thirumurthi U Shen J Xia W LaBaff AM Wei Y Li CW. MDM2-mediated degradation of SIRT6 phosphorylated by AKT1 promotes tumorigenesis and trastuzumab resistance in breast cancer. Sci Signal. (2014) 7:ra71. 10.1126/scisignal.2005076 25074979 PMC4161976

[30] Cho D Park H Park SH Kim K Chung M Moon W. The expression of DBC1/CCAR2 is associated with poor prognosis of ovarian carcinoma. J Ovarian Res. (2015) 8:2. 10.1186/s13048-015-0129-3 25823848 PMC4335761

[31] Kurman RJ International Agency for Research on Cancer, World Health Organization: WHO Classification of Tumours of Female Reproductive Organs. Lyon: International Agency for Research on Cancer (2014).

[32] Edge S AJCo Cancer: *AJCC Cancer Staging Handbook: From the AJCC Cancer Staging Manual*. New York, NY: Springer (2010).

[33] Therasse P Arbuck SG Eisenhauer EA Wanders J Kaplan RS Rubinstein L. New guidelines to evaluate the response to treatment in solid tumors. European Organization for Research and Treatment of Cancer, National Cancer Institute of the United States, National Cancer Institute of Canada. J Natl Cancer Inst. (2000) 92:205–16. 10.1093/jnci/92.3.205 10655437

[34] Allred D Harvey JM Berardo M Clark GM. Prognostic and predictive factors in breast cancer by immunohistochemical analysis. Mod Pathol. (1998) 11:155–68. 9504686

[35] Kim KM SH Park JS Bae SJ Noh GZ Tao JR Kim. FAM83H is involved in the progression of hepatocellular carcinoma and is regulated by MYC. Sci Rep. (2017) 7:3274. 10.1038/s41598-017-03639-3 28607447 PMC5468291

[36] Nelson WJ Nusse R. Convergence of Wnt, beta-catenin, and cadherin pathways. Science (2004) 303:1483–7. 10.1126/science.1094291 15001769 PMC3372896

[37] Jin J Zhan P Katoh M Kobayashi SS Phan K Qian H. Prognostic significance of beta-catenin expression in patients with non-small cell lung cancer: a meta-analysis. Transl Lung Cancer Res. (2017) 6:97–108. 10.21037/tlcr.2017.02.07 28331830 PMC5344847

[38] Zhang DP Li XW Lang JH. Prognostic value of beta-catenin expression in breast cancer patients: a meta-analysis. Asian Pac J Cancer Prev. (2015) 16:5625–33. 10.7314/APJCP.2015.16.14.5625 26320427

[39] Kim JR Moon YJ Kwon KS Bae JS Wagle S Yu TK. Expression of SIRT1 and DBC1 is associated with poor prognosis of soft tissue sarcomas. PLoS ONE (2013) 8:e74738. 10.1371/journal.pone.0074738 24019980 PMC3760851

[40] Bauer I Grozio A Lasiglie D Basile G Sturla L Magnone M. The NAD+-dependent histone deacetylase SIRT6 promotes cytokine production and migration in pancreatic cancer cells by regulating Ca2+ responses. J Biol Chem. (2012) 287:40924–37. 10.1074/jbc.M112.405837 23086953 PMC3510797

[41] Thiery JP Acloque H Huang RY Nieto MA. Epithelial-mesenchymal transitions in development and disease. Cell (2009) 139:871–90. 10.1016/j.cell.2009.11.007 19945376

[42] Iwatsuki M Mimori K Yokobori T Ishi H Beppu T Nakamori S. Epithelial-mesenchymal transition in cancer development and its clinical significance. Cancer Sci. (2010) 101:293–9. 10.1111/j.1349-7006.2009.01419.x 19961486 PMC11159985

[43] Xia YQ Hua RJ Juan C Zhong ZH Tao CS Fang R. SIRT6 Depletion sensitizes human hepatoma cells to chemotherapeutics by downregulating MDR1 expression. Front Pharmacol. (2018) 9:194. 10.3389/fphar.2018.00194 29563873 PMC5845756

[44] Ma SY Park JH Jung H Ha SM Kim Y Park DH. Snail maintains metastatic potential, cancer stem-like properties, and chemoresistance in mesenchymal mouse breast cancer TUBOP2J cells. Oncol Rep. (2017) 38:1867–76. 10.3892/or.2017.5834 28731185

[45] Witta SE Gemmill RM Hirsch FR Coldren CD Hedman K Ravdel L. Restoring E-cadherin expression increases sensitivity to epidermal growth factor receptor inhibitors in lung cancer cell lines. Cancer Res. (2006) 66:944–50. 10.1158/0008-5472.CAN-05-1988 16424029

[46] Bae JS Park JY Park SH Ha SH An AR Noh SJ. Expression of ANO1/DOG1 is associated with shorter survival and progression of breast carcinomas. Oncotarget (2018) 9:607–21. 10.18632/oncotarget.23078 29416639 PMC5787493

[47] Carey LA Sharpless NE. PARP and cancer–if it's broke, don't fix it. N Engl J Med. (2011) 364:277–9. 10.1056/NEJMe1012546 21208102 PMC3712751

[48] Mylona E Melissaris S Nomikos A Theohari I Giannopoulou I Tzelepis K. Effect of BRCA1 immunohistochemical localizations on prognosis of patients with sporadic breast carcinomas. Pathol Res Pract. (2014) 210:533–40. 10.1016/j.prp.2014.05.009 24947414

[49] Kim KM Moon YJ Park SH Park HJ Wang SI Park HS. Individual and combined expression of DNA damage response molecules PARP1, gammaH2AX, BRCA1, and BRCA2 predict shorter survival of soft tissue sarcoma patients. PLoS ONE (2016) 11:e0163193. 10.1371/journal.pone.0163193 27643881 PMC5028069

[50] Park SH Noh SJ Kim KM Bae JS Kwon KS Jung SH. Expression of DNA Damage response molecules PARP1, gammaH2AX, BRCA1, and BRCA2 predicts poor survival of breast carcinoma patients. Transl Oncol. (2015) 8:239–49. 10.1016/j.tranon.2015.04.004 26310369 PMC4562981

[51] Parkes EE Kennedy RD. Clinical application of poly(ADP-Ribose) polymerase inhibitors in high-grade serous ovarian cancer. Oncologist (2016) 21:586–93. 10.1634/theoncologist.2015-0438 27022037 PMC4861365

