# Supplementary material for: SIRT6 Is Involved in the Progression of Ovarian Carcinomas via β-Catenin-Mediated Epithelial to Mesenchymal Transition
Source: Front Oncol. 2018 Nov 20;8:538. doi: 10.3389/fonc.2018.00538 (PMC6256124; doi:10.3389/fonc.2018.00538)
Supplement: Supplementary file 1 [file Table_1.DOC]

Supplementary Material

# SIRT6 Is Involved in the Progression of Ovarian Carcinomas via β-Catenin-Mediated Epithelial to Mesenchymal Transition

**Jun Sang Bae^1^****^†^, Sang Jae Noh^2†^, Kyoung Min Kim****^1^, See-Hyoung Park^3^, Usama Khamis Hussein^1, 4^, Ho Sung Park^1^, Byung-Hyun Park****^5^, Sang Hoon Ha^6^, Ho Lee^2^, Myoung Ja Chung^1^, Woo Sung Moon^1^, Dong Hyu Cho^7*^, Kyu Yun Jang^1*^**

**^†^ These authors contributed equally to this work**

***Correspondence:**

Kyu Yun Jang

kyjang@chonbuk.ac.kr,

Dong Hyu Cho

obgy2001@jbnu.ac.kr

# Supplementary Table S1 Association between immunohistochemical expression of BRCA1, SIRT6, and active β-catenin in 104 ovarian carcinomas

| Markers |  | No. | BRCA1 |  |
| --- | --- | --- | --- | --- |
|  |  |  | Positive | *P* |
| Nu-SIRT6 | Negative | 68 | 18 (26%) | < 0.001 |
|  | Positive | 36 | 28 (78%) |  |
| Cy-SIRT6 | Negative | 55 | 16 (29%) | < 0.001 |
|  | Positive | 49 | 30 (61%) |  |
| Nu-Aβ-catenin | Negative | 48 | 12 (25%) | < 0.001 |
|  | Positive | 56 | 34 (61%) |  |
| Cy-Aβ-catenin | Negative | 45 | 11 (24%) | < 0.001 |
|  | Positive | 59 | 35 (59%) |  |

Nu-SIRT6; nuclear expression of SIRT6, Cy-SIRT6; cytoplasmic expression of SIRT6, Nu-Aβ-catenin; nuclear expression of active β-catenin, Cy-Aβ-catenin; cytoplasmic expression of active β-catenin
